# Influence of Nivolumab for Intercellular Adhesion Force between a T Cell and a Cancer Cell Evaluated by AFM Force Spectroscopy

**DOI:** 10.3390/s20195723

**Published:** 2020-10-08

**Authors:** Hyonchol Kim, Kenta Ishibashi, Masumi Iijima, Shun’ichi Kuroda, Chikashi Nakamura

**Affiliations:** 1 Cellular and Molecular Biotechnology Research Institute, National Institute of Advanced Industrial Science and Technology (AIST), Tsukuba 305-8565, Japan; chikashi-nakamura@aist.go.jp; 2 Graduate School of Engineering, Tokyo University of Agriculture and Technology, Tokyo 184-8588, Japan; kenta.ishibashi@miraca.com; 3 Department of Nutritional Science and Food Safety, Faculty of Applied Bioscience, Tokyo University of Agriculture, Tokyo 156-8502, Japan; mi206786@nodai.ac.jp; 4 Department of Biomolecular Science and Reaction, The Institute of Scientific and Industrial Research, Osaka University, Osaka 567-0047, Japan; skuroda@sanken.osaka-u.ac.jp

**Keywords:** atomic force microscopy, nivolumab, PD-1, PD-L1, cell adhesion, microcup

## Abstract

The influence of nivolumab on intercellular adhesion forces between T cells and cancer cells was evaluated quantitatively using atomic force microscopy (AFM). Two model T cells, one expressing high levels of programmed cell death protein 1 (PD-1) (PD-1^high^ Jurkat) and the other with low PD-1 expression levels (PD-1^low^ Jurkat), were analyzed. In addition, two model cancer cells, one expressing programmed death-ligand 1 (PD-L1) on the cell surface (PC-9, PD-L1^+^) and the other without PD-L1 (MCF-7, PD-L1^−^), were also used. A T cell was attached to the apex of the AFM cantilever using a cup-attached AFM chip, and the intercellular adhesion forces were measured. Although PD-1^high^ T cells adhered strongly to PD-L1^+^ cancer cells, the adhesion force was smaller than that with PD-L1^−^ cancer cells. After the treatment of PD-1^high^ T cells with nivolumab, the adhesion force with PD-L1^+^ cancer cells increased to a similar level as with PD-L1^−^ cancer cells. These results can be explained by nivolumab influencing the upregulation of the adhesion ability of PD-1^high^ T cells with PD-L1^+^ cancer cells. These results were obtained by measuring intercellular adhesion forces quantitatively, indicating the usefulness of single-cell AFM analysis.

## 1. Introduction

Understanding of the interaction between cancer cells and immune cells is an important topic in anticancer therapy. For example, it is known that a portion of cancer cells survive attacks by T cells using an immune check-point system, and one key mediator of this interaction occurs between programmed cell death protein 1 (PD-1), expressed on the surface of T cells, and programmed death-ligand 1 (PD-L1), a ligand of PD-1 expressed on cancer cells [1,2,3,4]. Inhibition of the interaction between PD-1 and PD-L1 is a target of cancer therapy, and nivolumab is a well-known antibody drug that inhibits this interaction [5,6]. One approach to understand the mechanism of the immune check-point system with multilateral points of view is the measurement of changes in adhesion strength between T cells and cancer cells before and after the administration of nivolumab; however, such an evaluation has never been carried out.

Atomic force microscopy (AFM) is a powerful tool to measure the mechanical properties of single living cells. The AFM tip can approach the cell surface; therefore, stiffnesses (Young’s moduli) of cancer cells were quantitatively measured by pushing the cell surface with the AFM tip, and they were discussed with a view point of the metastatic abilities of the cancer cells [7,8,9]. The electrochemical activities of cardiac cells can be also measured directly by using scanning electrochemical microscopy to evaluate the activities of the cells [10,11]. The interaction forces of various target pairs, such as binding between ligands and their specific receptors, were measured on cell surfaces with immobilizations of the ligands to the AFM tip surfaces [12,13,14,15,16,17,18,19,20]. AFM can also be applied for the measurement of intercellular adhesion forces [8,21,22,23,24,25,26,27], and, in that case, a cell should be attached to the apex of the AFM cantilever. In our previous studies, a microcup with almost the same diameter as that of a cell [28] was attached to the AFM cantilever. The cup-attached chip (cup-chip) was used to approach a cell; the cell was trapped in the cup and picked up within a few seconds; and the intercellular adhesion forces were measured quantitatively [8,25,26,27]. In this study, the same method was applied for the measurement of interaction forces between T cells and cancer cells, and during the measurement, nivolumab was administered to the measurement system to evaluate the influence of nivolumab on intercellular adhesion forces.

## 2. Materials and Methods

### 2.1. Cell Lines and Analysis of Gene Expression

In this study, four cell lines were used as a model for the measurement of the interaction forces between T cells and cancer cells: (i) human T cells on which PD-1 molecules were highly expressed (PD-1 effector Jurkat cells, Promega, USA; hereafter PD-1^high^), (ii) human T cells on which PD-1 molecules were expressed at a low level (Jurkat, RIKEN BRC, Japan; hereafter PD-1^low^), (iii) human lung adenocarcinoma cancer cells on which PD-L1 molecules were highly expressed (PC-9, RIKEN BRC; hereafter PD-L1^+^), and (iv) human breast cancer cells on which PD-L1 molecules were not expressed (MCF-7, JCRB Cell Bank, Osaka, Japan; hereafter PD-L1^−^). All cell lines were cultivated in commercially available cell culture media (RPMI 1640, Sigma Aldrich, St. Louis, MO, USA) containing 10% fetal bovine serum (Gibco, Thermo Fisher Scientific, Waltham, MA, USA) at 37 °C in 5% CO_2_. For the measurement of intercellular interaction forces using AFM, cancer cells (either PD-L1^+^ or PD-L1^−^) were cultivated in a 35-mm cell culture dish at a concentration of 5 × 10^4^ cells, and used in an adherent state on the dish bottom.

### 2.2. Immunofluorescence Assays

The expression levels of PD-1 on T cells and PD-L1 on cancer cells were evaluated using immunofluorescence assays. The T cells (PD-1^high^ and PD-1^low^) were non-adherent; therefore, the cells were immobilized on a glass substrate using lipid bilayer-anchoring reagent (oleyl-O(CH_2_CH_2_)_n_CO–CH_2_CH_2_–COO–N-hydroxysuccinimide, NOF, Tokyo, Japan) as in our previous study [20], and immunofluorescence assays were performed as for adherent cells. Briefly, 1 × 10^4^ cells were treated with 4% paraformaldehyde (Fujifilm Wako Pure Chemical, Osaka, Japan) in phosphate-buffered saline (PBS) for 10 min. After washing with PBS, the cells were reacted with 2 µg/mL of either anti-PD-1 antibody (Thermo Fisher Scientific, for T cells) or anti-PD-L1 antibody (Cell Signaling Technology, Danvers, MA, USA, for cancer cells) in PBS containing 0.4% blocking reagent composed of skim milk (BlockAce, DS Pharma Biomedical, Osaka, Japan) for 1 h at room temperature. After washing the cells with PBS, the cells were treated with secondary antibodies conjugated with fluorescent dyes (Alexa Fluor 488-labeled anti-mouse IgG (for PD-1) and anti-rabbit IgG (for PD-L1) monoclonal antibodies, Thermo Fisher Scientific) at a concentration of 2 µg/mL in PBS containing 0.4% blocking reagent for 1 h at room temperature. After washing the cells with PBS, observations were performed using either a confocal fluorescence microscope (FV300, Olympus, Tokyo, Japan) or a general fluorescence microscope combined with a cooled CCD camera (DP30, Olympus).

### 2.3. Cell Pick-Up and Force Measurements

The cup-chip was prepared as reported previously [25]. Briefly, microcups to attached to the AFM cantilever were fabricated using polystyrene particles (diameter, 10 µm, coefficient of variation, 1.20%, JSR, Japan) as templates with a thermal deposition method [28]. A fabricated microcup was attached to the apex of an AFM cantilever (DNP, Bruker, Billerica, MA, USA) using micromanipulators (MN-4 and MMO-202ND, Narishige, Tokyo, Japan). The typical values of the spring constants of the cantilevers were 0.06 N/m, which was calibrated by the thermal fluctuation method for each cantilever.

Cell pick-up and force measurements were performed using an AFM combined with an inverted optical microscope (NanoWizard II, JPK Instruments, Berlin, Germany) in cell culture media at room temperature. In this study, a T cell (either PD-1^high^ or PD-1^low^) was captured in the cup and was used to approach a cancer cell (either PD-L1^+^ or PD-L1^−^) adhered to the dish bottom. To make the holding of the T cell to the inner concave of the cup tight enough, the cup-chip was immersed in 0.01% poly-L-lysine (Sigma Aldrich) solution for 5 min to coat the cup surfaces with poly-L-lysine molecules and dried.

The cell pickup and force measurements were carried out similarly to our previous studies [8,25,26,27]. Firstly, a small glass substrate on which polytetrafluoroethylene (PTFE) was coated was placed in a 35-mm cell culture dish on which cancer cells were still adherent, and 50 µL of T cell (either PD-1^high^ or PD-1^low^) suspension containing 1 × 10^4^ cells was dropped onto the PTFE substrate. Next, cell culture medium was poured into the dish to fill the dish with medium. The poly-L-lysine-coated cup-chip was used to approach a T cell using the stepping motor of the instrument, retracted after a few seconds. Then the T cell was captured in the cup-chip. After T cell capture, the cell was set towards the top of a cancer cell (either PD-L1^+^ or PD-L1^−^) adhering to the dish bottom, and measurements of the intercellular adhesion forces were performed. Continuous holding of the T cell in the cup was confirmed using optical microscope observations combined with AFM throughout all measurements. The conditions of the force curve measurements were as follows: 100 µm *Z* scan size; 5 µm/s *Z* scan velocity; 1 nN initial loading force; and 0, 5, 10, 20, 30, or 60-s dwell time at the two cells’ contact with a constant height mode. Seven PD-1^high^ T cells and four PD-1^low^ T cells were picked up, and the measurements were performed six times on each PD-L1^+^ cancer cell in maximum with six different dwell times. The force curve measurements were carried out on 44 and 32 PD-L1^+^ cancer cells in total using PD-1^high^ and PD-1^low^ T cells, respectively (*N* = 24, 25, 27, 24, 25, and 26 for PD-1^high^ vs. PD-L1^+^ and 17, 22, 24, 21, 21, and 21 for PD-1^low^ vs. PD-L1^+^ at 0, 5, 10, 20, 30, and 60-s dwell times, respectively). The same force curve measurements were also carried out using T cells (PD-1^high^ and PD-1^low^) and PD-L1^−^ cancer cells (*N* = 15 for all cases, respectively).

### 2.4. Measurements of Intercellular Adhesion Forces in the Presence of Nivolumab

For the evaluation of the change in intercellular adhesion forces between T cells and cancer cells by the addition of an antibody drug, T cells were treated with nivolumab before the pick-up of cells with the cup-chip. In detail, 1 × 10^4^ of T cells (PD-1^high^ and PD-1^low^) were suspended with culture media containing 5 µg/mL of nivolumab (OPDIVO^®^, Ono Pharmaceutical, Japan) and incubated for 30 min at 37 °C in 5% CO_2_. After washing the T cells with culture media, the nivolumab-treated T cells were captured on the cup-chip, and force curve measurements were performed as described in Section 2.3. (*N* = 17, 18, 18, 18, 18, and 18 for PD-1^high^ vs. PD-L1^+^ and 6, 7, 8, 8, 8, and 8 for PD-1^low^ vs. PD-L1^+^ at 0, 5, 10, 20, 30, and 60-s dwell times, respectively).

## 3. Results

**Figure 1:**
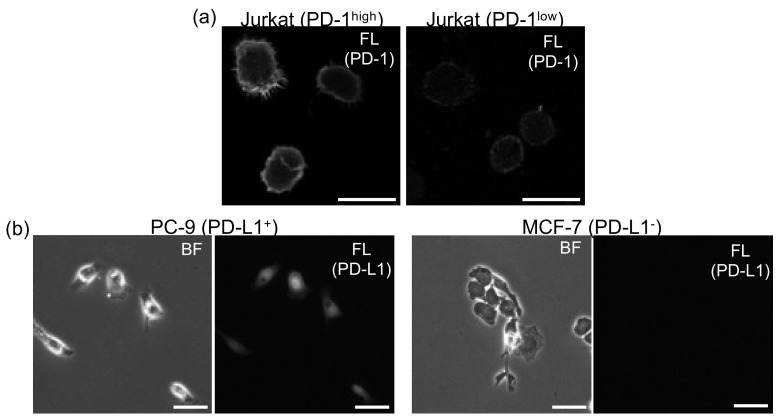
Immunofluorescent staining of PD-1 molecules on T cells (**a**) and PD-L1 molecules on cancer cells (**b**) used in this study. BF, bright field; FL, fluorescence images. Bars, 50 µm.

The expression levels of both PD-1 on T cells and PD-L1 on cancer cells were evaluated using immunofluorescence assays. Figure 1 shows results of the assays. Firstly, the expression levels of PD-1 between PD-1^high^ and PD-1^low^ T cells were compared (Figure 1a). Although PD-1 molecules were expressed on the PD-1^low^ cell surface, the expression level on PD-1^high^ cells was relatively much higher than that on PD-1^low^ cells. Next, the expression levels of PD-L1 molecules were evaluated for two cancer cell lines, PC-9 and MCF-7 (Figure 1b). As shown in the figure, the expression on PC-9 cells was relatively much higher than that on MCF-7 cells. Therefore, we used PC-9 cells as model cancer cells expressing PD-L1 molecules (PD-L1^+^) and MCF-7 as those not expressing PD-L1 (PD-L1^−^), which was also consistent with a previous report [29]. In this study, the intercellular adhesion forces between a PD-L1^+^ cell and the two T cells (PD-1^high^ and PD-1^low^) were mainly measured to evaluate the contribution of PD-1/PD-L1 interactions to intercellular adhesion strengths between T cells and cancer cells, and the influence of nivolumab on adhesion strength was evaluated.

**Figure 2:**
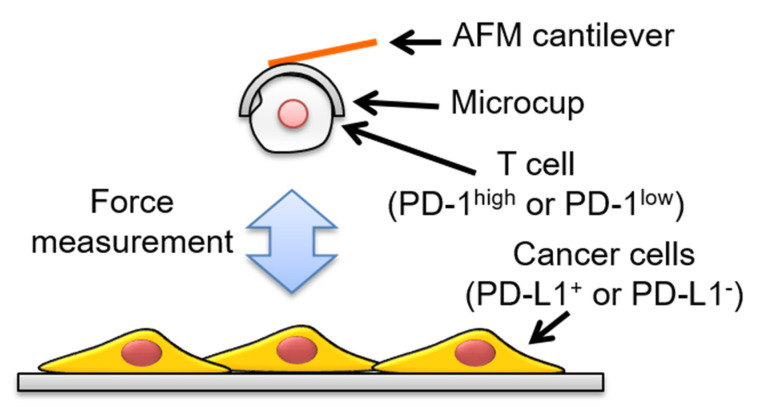
Experimental setup used in this study. A T cell was picked up using the cup-chip, used to approach a cancer cell, and the intercellular adhesion force was measured.

**Figure 3:**
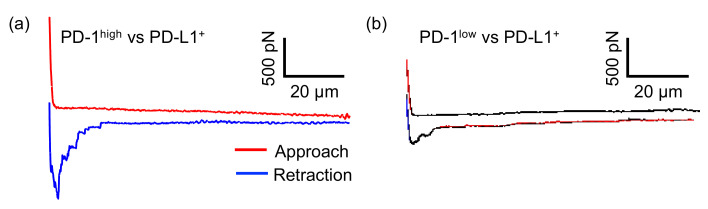
Typical force curves obtained between PD-L1^+^ cancer cells and either PD-1^high^ (**a**) or PD-1^low^ (**b**) cell pairs. Dwell time, 20 s.

**Figure 4:**
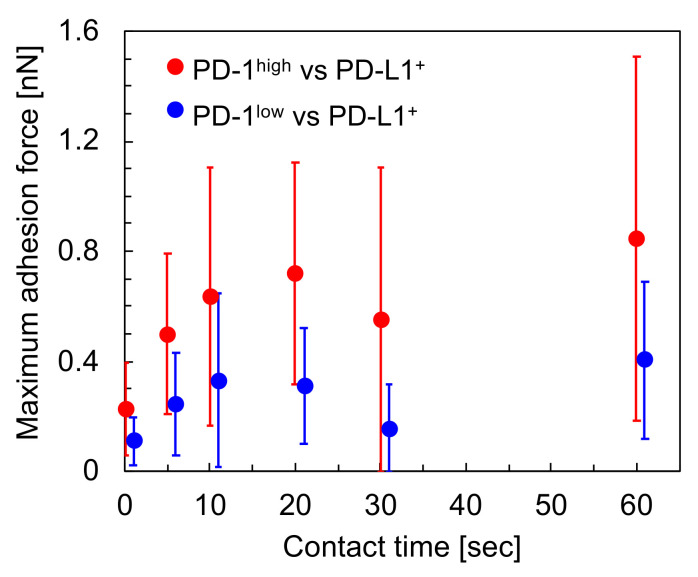
Relationship between maximum adhesion forces and contact time between PD-L1^+^ cancer cells and either PD-1^high^ (red) or PD-1^low^ (blue) T cells. Data points were a few shifted to left and right for visualization. *p* < 0.01 for all contact time.

Figure 2 shows a schematic image of the experimental setup used in this study to measure intercellular adhesion forces [25]. In this experiment, a T cell (either PD-1^high^ or PD-1^low^) was picked up and used to approach a cancer cell (either PD-L1^+^ or PD-L1^−^). For T cell pick-up, the cup-chip was used to approach a T cell, incubated for a few seconds, and retracted, and the cell was captured in the cup. The cell-attached chip was moved towards a cancer cell adhering to a substrate, and force curve measurements were carried out to evaluate the intercellular adhesion forces between the T cell and the cancer cell. Figure 3 shows typical force curves obtained between PD-L1^+^ cancer cells and either PD-1^high^ (Figure 3a) or PD-1^low^ (Figure 3b) cell pairs. Intercellular adhesion forces, which can be detected as the bending of the AFM cantilever toward the substrate and represented as large peaks in the retraction curves, were observed as curves for both cell pairs, as shown in Figure 3. These measurements were repeated using individual cancer cells with various contact times, and maximum adhesion forces were calculated from each curve in the same manner as in a previous study [8]. Figure 4 shows a summary of the average values of the maximum adhesion forces between PD-L1^+^ cancer cells and either PD-1^high^ or PD-1^low^ cell pairs against the contact times of the cells. We found that the force between PD-1^high^ T cells and PD-L1^+^ cancer cells was stronger than that between PD-1^low^ T cells and PD-L1^+^ cancer cells for all contact times (*p* < 0.01), indicating that T cells on which PD-1 was highly expressed adhered strongly to cancer cells on which PD-L1 was expressed and that there is a possibility that the interaction between PD-1 and PD-L1 contributes to the adhesion between the T cell and the cancer cell.

**Figure 5:**
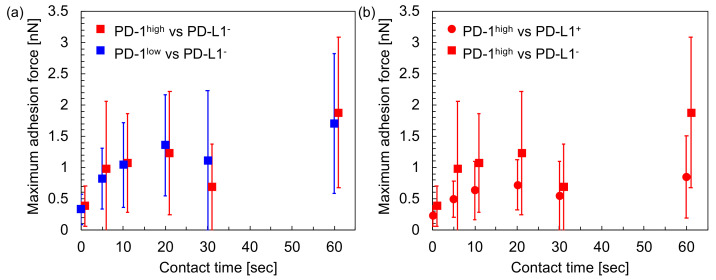
Summary of the intercellular adhesion force of T cells to PD-L1^−^ cancer cells. (**a**) Relationship between maximum adhesion forces and the contact time between PD-L1^−^ cancer cells and either PD-1^high^ (red) or PD-1^low^ (blue) T cells. *p* > 0.05 for all contact times. (**b**) Comparison of the adhesion force of PD-1^high^ T cells to PD-L1^+^ (circle, same data in Figure 4) and PD-L1^−^ (square) cancer cells. Data points were shifted to left and right for visualization. *p* < 0.05 for all contact time.

For the evaluation of the above contribution, i.e., the contribution of the interaction between PD-1 and PD-L1 for intercellular adhesion, we also measured the intercellular adhesion forces between a T cell (either PD-1^high^ or PD-1^low^) and a PD-L1^−^ cancer cell. Figure 5a shows the summary of the average values of the maximum adhesion forces against the contact times of the cell pairs. As shown in the figure, there was no significant difference between PD-1^high^ and PD-1^low^ cells for all contact times (*p* > 0.05). As a noteworthy point, the intercellular adhesion force between PD-1^high^ and PD-L1^−^ was larger than that between PD-1^high^ and PD-L1^+^ for all contact times (*p* < 0.05, Figure 5b). The results in Figure 5 suggest that the interaction between PD-1 and PD-L1 contributes to the adhesion between T cells and cancer cells, as shown in Figure 4; however, the contribution is limited and is not the dominant factor for adhesion, and the cancer cells on which PD-L1 was not expressed still adhered strongly to the T cells in comparison with the PD-L1-positive cancer cells.

**Figure 6:**
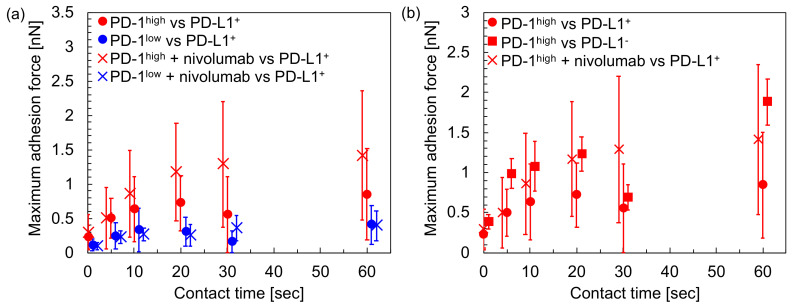
Summary of the influence of nivolumab on intercellular adhesion forces between T cells and cancer cells. (**a**) Comparison of the intercellular adhesion forces between T cells (either PD-1^high^ or PD-1^low^) and PD-L1^+^ cancer cells before (circle, same data in Figure 4) and after (cross) treatment with nivolumab. For PD-1^high^ vs. PD-L1^+^ before and after the treatment of nivolumab, *p* < 0.05 over 20 s contact time. (**b**) Comparison of the adhesion force of PD-1^high^ T cells to PD-L1^+^ (circle, Figure 4), PD-L1^−^ (square, Figure 5), and PD-L1^+^ cancer cells after treatment with nivolumab (cross). *p* > 0.05 between PD-L1^−^ (square) and PD-L1^+^ after treatment with nivolumab (cross) for all contact times. Data points were shifted to left and right for visualization.

To evaluate the influence of antibody drugs on the intercellular adhesion between T cells and PD-L1^+^ cancer cells, T cells (PD-1^high^ and PD-1^low^) were treated with nivolumab, an antibody drug targeting PD-1 molecules on T cell surfaces. The treated T cells were picked up using the cup-chip, and force curve measurements were performed with PD-L1^+^ cancer cells. Figure 6a shows the comparison of the intercellular adhesion forces before and after treatment of the T cells with nivolumab. After the treatment of PD-1^high^ cells with nivolumab, the intercellular adhesion force with PD-L1^+^ cells increased (*p* < 0.05 over 20 s). The increase in intercellular adhesion force with the addition of nivolumab was not significant between PD-1^low^ and PD-L1^+^ cells (*p* > 0.05), suggesting that nivolumab promoted the intercellular adhesion between T cells highly expressing PD-1 and cancer cells expressing PD-L1. After the treatment of PD-1^high^ cells with nivolumab, the intercellular adhesion forces increased at similar level to those between PD-1^high^ and PD-L1^−^ cells (*p* > 0.05), as summarized in Figure 6b.

## 4. Discussion

In this study, the intercellular adhesion forces between T cells and cancer cells were measured quantitatively using AFM with a focus on the interaction between PD-1 and PD-L1. According to our results, the following relationships were found: (i) adhesion between PD-1^high^ T cells and PD-L1^+^ cancer cells was stronger than that between PD-1^low^ T cells and PD-L1^+^ cancer cells (Figure 4); (ii) PD-1^high^ T cells adhered to PD-L1^−^ cancer cells more strongly in comparison to PD-L1^+^ cancer cells (Figure 5); and (iii) the adhesion force between PD-1^high^ T cells and PD-L1^+^ cancer cells increases to a similar level to that between PD-1^high^ T cells and PD-L1^−^ cancer cells using the treatment of T cells with nivolumab (Figure 6). These results suggested that, firstly, although the interaction between PD-1 and PD-L1 partially contributed to the intercellular adhesion between T cells and cancer cells, the interaction might be not a dominant factor for adhesion and, secondly, nivolumab may affect the upregulation the adhesion ability of T cells with cancer cells expressing PD-L1 molecules. It is known that a portion of malignant cancer cells avoid attack by immune cells by expressing PD-L1 molecules [1,2,3,4] and that, for the attack of cancer cells by immune cells, sufficient interactions, such as long-term contact mediated with strong intercellular adhesions, between cancer cells and immune cells might be required. Nivolumab molecules affect the immune check-point system mediated by PD-1/PD-L1 interactions. According to our results, one assumption may be that, firstly, PD-1 on T cells might mediate the recognition of cancer cells expressing PD-L1; however, key molecules mediating the intercellular adhesion may not be PD-1/PD-L1 but other molecules. Secondly, the expression of PD-L1 on cancer cells might reduce the adhesion strength for T cells, and, thirdly, the adhesion forces of T cells expressing PD-1 with cancer cells expressing PD-L1 might recover through the influence of nivolumab, which might help the attack of T cells on malignant cancer cells. The detailed molecular mechanism behind the increase in intercellular adhesion force was still unclear, i.e., whether the increase was achieved by the changes in T cell phenotypes with the stimulation of nivolumab, such as by the regulation of the unknown key adhesion mediators, or by changes in the physical properties of the T cell surfaces with the adsorption of nivolumab molecules. However, it was clearly and quantitatively evidenced that the intercellular adhesion force between PD-1^high^ T cells and PD-L1^+^ cancer cells increased with the treatment of the T cells with nivolumab. Although our results were obtained using model cell lines in vitro, these findings were firstly obtained by measuring intercellular adhesion forces quantitatively, indicating the usefulness of the experimental approach suggested in this study.

As shown in Figure 4, average values of the maximum adhesion forces between PD-1^high^ T cell and PD-L1^+^ cancer cell were less than 1 nN; even when the contact time was 60 s, which was 0.3 to 0.8 times that in our previous studies [26]. Though the actual reason why the forces were small in comparison with those in previous studies was not clear, one possibility might be that the T cells were non-adherent cells. In our previous studies, we measured cellular stiffnesses including T cells [20], and the variety of the stiffnesses was smaller than the difference of maximum adhesion forces between this study and previous studies. Therefore, we concluded that relatively small force values did not originate in the difference of contact area between these studies but originated in the difference in the phenotypes of the cells.

In this study, 1 nN of loading force was applied to the cells. The value of the loading force was determined as follows: (i) enough contact area should be maintained at the two cells’ contact, and obtained force values represent the strength required to detach of the cell pair, not to break a single or a few molecular bonds on the cell surfaces; and (ii) damage to the cells should be small enough. The average value of contact area in this study was estimated as about 16 µm^2^, which corresponded to about 10% of the area of the hemispherical cell surface with an assumption of the cellular diameter as 10 µm. The cells even survived force measurements that were repeated several times on a cell. These results indicate that the loading force used in this study satisfies the above two requirements. As shown in Figure 4, Figure 5 and Figure 6, the values of standard deviations were large. This might originate in the heterogeneity of individual cells and not in the small contact area because the coefficients of the variations in the measurements of cell adhesion forces were more than 50%, even as the contact area increased with the increase of loading forces [20].

## 5. Conclusions

In this study, intercellular adhesion forces between T cells and cancer cells were measured quantitatively using AFM spectroscopy. Two model T cells, PD-1^high^ and PD-1^low^, and two model cancer cells, PD-L1^+^ and PD-L1^−^, were prepared; any one of the T cells was picked up using a cup-attached AFM chip, which approached one of the cancer cells, and the intercellular adhesion forces were measured. As a result, (i) PD-1^high^ T cells adhered to PD-L1^+^ cancer cells more strongly in comparison with PD-1^low^ T cells; (ii) PD-1^high^ T cells adhered to PD-L1^−^ cancer cells more strongly in comparison with PD-L1^+^ cancer cells; and (iii) the adhesion force between PD-1^high^ T cells and PD-L1^+^ cancer cells was increased by the treatment of the T cells with nivolumab. These results may be explained: firstly, the interaction between PD-1 on T cells and PD-L1 on cancer cells partially contributes to intercellular adhesion; however, the interaction was not a dominant factor for cell adhesion. Secondly, nivolumab influenced the upregulation of the adhesion ability of PD-1^high^ T cells with PD-L1^+^ cancer cells. Though the detailed molecular mechanisms for the obtained results were not clear, possible explanations may be that, firstly, PD-1 on T cells might mediate the recognition of cancer cells expressing PD-L1; however, tight adhesion of the two cells might be mediated by other molecules. Secondly, the expression of PD-L1 on cancer cells might reduce the adhesion strength for T cells, and, thirdly, the adhesion forces of T cells expressing PD-1 with cancer cells expressing PD-L1 might recover through the influence of nivolumab, which might help the attack of T cells on malignant cancer cells. These results were obtained by measuring the intercellular adhesion forces quantitatively, indicating the usefulness of the AFM spectroscopy suggested in this study.
